# Higher-Order Spectrum in Understanding Nonlinearity in EEG Rhythms

**DOI:** 10.1155/2012/206857

**Published:** 2012-02-08

**Authors:** Cauchy Pradhan, Susant K. Jena, Sreenivasan R. Nadar, N. Pradhan

**Affiliations:** ^1^Medical Image Processing Lab, EPFL, 1015 Lausanne, Switzerland; ^2^Department of Computer Science and Mathematics, K.N.S. Institute of Technology, Bangalore 560064, India; ^3^MEG Core, National Institute of Mental Health, Bethesda, MD 20892, USA; ^4^Department of Psychopharmacology, NIMHANS, Bangalore 560029, India

## Abstract

The fundamental nature of the brain's electrical activities recorded as electroencephalogram (EEG) remains unknown. Linear stochastic models and spectral estimates are the most common methods for the analysis of EEG because of their robustness, simplicity of interpretation, and apparent association with rhythmic behavioral patterns in nature. In this paper, we extend the use of higher-order spectrum in order to indicate the hidden characteristics of EEG signals that simply do not arise from random processes. The higher-order spectrum is an extension Fourier spectrum that uses higher moments for spectral estimates. This essentially nullifies all Gaussian random effects, therefore, can reveal non-Gaussian and nonlinear characteristics in the complex patterns of EEG time series. The paper demonstrates the distinguishing features of bispectral analysis for chaotic systems, filtered noises, and normal background EEG activity. The bispectrum analysis detects nonlinear interactions; however, it does not quantify the coupling strength. The squared bicoherence in the nonredundant region has been estimated to demonstrate nonlinear coupling. The bicoherence values are minimal for white Gaussian noises (WGNs) and filtered noises. Higher bicoherence values in chaotic time series and normal background EEG activities are indicative of nonlinear coupling in these systems. The paper shows utility of bispectral methods as an analytical tool in understanding neural process underlying human EEG patterns.

## 1. Introduction

Biological signals are highly complex and understandably nonlinear, may it be the firing of neurons, the beating of the heart, or breathing. The nature of the signals is dramatic and appears to be esoteric. These signals arise out of a multitude of interconnected elements comprising of the human body. These are bounded, finite, and the connections are weakly coupled across all elements. These vary in time scales ranging from nanoseconds for molecular motion to gross observable behavior in terms of days and years. This implies that biological systems are nonstationary [1, 2]. Signals of biological origin require considerable experience for their analysis and interpretation. This is only gained through practical training and hand on expertise as most signals are contaminated by processes of nonbiological origin at the recording stage. Further, these signals can be mimicked by noise and artifacts [3]. One of the contentious issues is that filtered noises mimick time series of brain electrical potentials which substantially vitiate both extraction of linear and nonlinear measures of these time series.

Electroencephalography (EEG) is the recording of brain cortical electrical activity from electrodes placed on the scalp. The signals are also recorded subdurally or directly on the cortex and are called Electrocorticogram (ECoG). The dynamics and the structure patterns of both EEG and ECoG are similar. EEG signals are used in seizure detection, organic encephalopathies, monitoring anesthesia, and for the determination of brain death. There are perhaps 10^5^ neurons under each square millimeter of the cortical surface. Scalp EEG measures space-averaged activity of 10^7^ or more neurons implying a source area of at least a square centimeter. EEG signals are quasistationary not exceeding 2–4 seconds in eye-open state or 20–30 seconds in deep sleep. The stationary condition varies with fleeting attention, and thus analytical methods based on the assumption of the stationarity of the system are superfluous. Signal prediction, therefore, is fallacious other than the mere detection of trends. Under these circumstances, linear stochastic models remain the primary method for the analysis of biological time series due to their robustness, simplicity of interpretation, and their apparent association with rhythmic behavioral patterns in nature. The Fourier spectrum is trivial to linear methods. The linear algebraic techniques and spectral estimates over years have shown good correlation as approximation for inherently nonlinear functions that are biological data [4–7].

Over decades of research publications, the application of nonlinear time series methods involving invariant measures such as Correlation Dimension (D_2_), Lyapunov Exponents (*λ*), and Fractal Dimension (D_1_), have not gained wide acceptance in biomedical field. The measures do not provide real insight into the biological phenomena due to their inherent high dimensionality along with long- and short-range interactions within these systems [8–12]. The short data lengths of biological time series deny perfect reconstruction of their attractor. In attractor reconstruction, true independence of each vector is not guaranteed due to the partial correlations across dimensions. Therefore, nonlinear invariant measures like D_2_, D_1_, and *λ* have limited value in medicine. Even if one establishes the presence of chaos in deep sleep or anesthesia [8–12], it is very difficult to reconstruct a model that can reproduce the time series reflecting the behavioral transitions during sleep and their apparent associations with the time series. More so, the filtration of WGN using stiff linear filters can mimic chaotic process including its validity by surrogate testing [3, 13–16]. The results of nonlinear time series invariants could not be validated as two different persons in the same behavioral states have varying values. We have reported D_2_ values in the range of 2–4 in seizures and deep sleep. Similar D_2_ values are also found in normal healthy individuals in awake state [17–22]. Therefore, the biological acceptability of published nonlinear time series measures is low. Even if we get a long-range pattern, there remain high degrees of overlap in such invariant measures across behavioral states.

In order to elicit better inference of EEG time series data, we have extended the Fourier transform to bispectral estimation using higher-order (third) moment [23–26]. This nullifies all Gaussian random effects in the process. While bispectrum analysis detects nonlinear interactions, it does not quantify the coupling strength which we have evaluated through bicoherence. The bicoherence, that is, a normalized bispectrum has been used in sleep studies in animals [27]. There are also several reports of its application to human brain signals, mainly, monitoring the degree of consciousness or depth of anaesthesia. Bispectral methods have led to the development of a device (Bispectral Index Monitor) which has been recommended for clinical monitoring in critical care and surgical anesthesia [28]. Recently, bispectral measures have been extended to detect subtle changes in EEG dynamics in visual representation of motor tasks [29]. However, application of higher-order spectral techniques is less wide-spread in medicine and biology. The aim of the present investigation is to demonstrate the utilities of higher-order spectrum in human EEG processing. There is a need to represent the standard rhythmic manifestations of EEG as revealed by bispectrum and reinforce its clinical and research applications. All descriptions of brain electrical activity recordings invariably refer to these well-established background EEG rhythms. This paper also demonstrates the significance of nonlinear interactions and coupling of brain activity in the cerebral cortex using background EEG activities of normal subjects. We have used delta, theta, alpha, beta, and indeterminate activities for the above study and contrasted the results with chaotic time series (Lorenz and Mackey-Glass systems), white Gaussian noise, and filtered noises (1.5 Hz, 3 Hz, 9 Hz, 30 Hz, and 300 Hz).

## 2. Method

Any process is a linear process with respect to its second-order statistics (power spectrum). The autocorrelation sequence does not give any evidence of nonlinearity. In contrast, higher-order cumulants can give evidence of nonlinearity (i.e., bispectrum for quadratic interactions). Polyspectrum estimators are the natural generalizations of the autocorrelation function, and cumulants are specific nonlinear combinations of these moments. The power spectrum does not carry information about phase which can be recovered from higher-order polyspectra. The use of higher-order moments nullifies all Gaussian random effects of the process, and the bicoherence can then quantify the degree of the remaining nonlinear coupling. The bispectrum and its normalized derivative, the bicoherence, describes the components of a time series that deviates from a Gaussian amplitude distribution. Bispectra have been used to examine various physical time series data including plasma physics and ocean waves [23–26].

Fourier transform of *f*(*t*) is given by

(1) $$f^{\prime }\left(w\right)=\overset{\infty }{\underset{-\infty }{\int }}f\left(t\right)e^{-i\omega t}dt.$$

Power spectrum of (1) is

(2) $$Pf\left(w\right)={\left|f^{\prime }\left(w\right)\right|}^{2}.$$

The natural estimate of the bispectrum (*B*_*xxx*_) is the Fourier transform of the third-order cumulant sequence (*C*_*xxx*_):

(3) $$B_{xxx}\left(f_{1},f_{2}\right)=\overset{\infty }{\underset{k=-\infty }{\sum }}\overset{\infty }{\underset{l=-\infty }{\sum }}C_{xxx\;}\left(k,l\right)e^{-i2\pi f_{1}k}\;e^{-i2\pi \;f_{2}l}.$$

The bispectrum can be also written as (4), where *X*_*n*_ is the Fourier Transform of {*X*_*n*_}:

(4) $$B_{xxx}\left(f_{1},f_{2}\right)=\frac{1}{N^{2}}\;X_{n^{\prime }}\;\left(f_{1}+f_{2}\right)\;X_{n}f_{1}X_{n}f_{2}.$$

The bicoherence or the normalized bispectrum (5) is a measure of the amount of phase coupling that occurs in a signal or between two signals. Phase coupling is the estimate of the proportion of energy in every possible pair of frequency components, *f*_1_, *f*_2_ (i.e., 1–50 Hz in EEG), which satisfies the definition of quadratic phase coupling (phase of component at *f*_3_, which is *f*_1_ + *f*_2_, equals phase of *f*_1_+ phase of *f*_2_) [23, 25]:

(5) $$\text{bic}\left(f_{1},\;f_{2}\right)=\frac{{\left|B\left(f_{1},f_{2}\right)\right|}^{2}}{P\left(f_{1}\right)P\left(f_{2}\right)P\left(f_{1}+f_{2}\right)}.$$

When the analyzed signal exhibits structure of any kind whatsoever, it might be expected that some phase coupling occurs. Bicoherence analysis is able to detect coherent signals in extremely noisy data, provided that the coherency remains constant for sufficiently long times, since the noise contribution falls off rapidly with increasing *N*. The bicoherence due to the coherent signal should be at least a factor of 3 above the maximum noise contribution, and the allowable signal-to-noise ratio is 3e. Bicoherence can be considered a very powerful noise filter. We have utilized the archived EEG data available in the Department of Psychopharmacology at the National Institute of Mental Health and Neurosciences, Bangalore. We have taken normative data of standard frequency band of EEG patterns of alpha, beta, theta, delta and indeterminate activities. These pattern descriptions are the accepted background rhythms in the EEG literature.

## 3. Results

**Figure 1:**
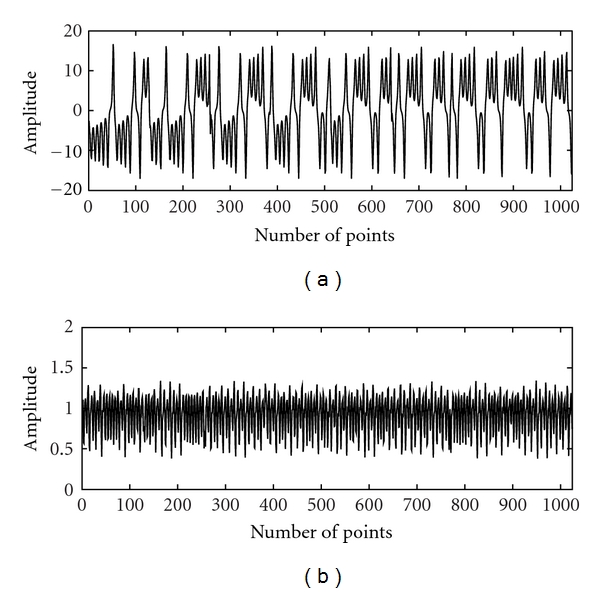
The time series of the (a) Lorenz (*x*-component) and (b) the Mackey-Glass systems. For each, 200000 data points were generated of which first 5000 data points were discarded to remove the initial transients. Only 1024 data points are shown here.

**Figure 2:**
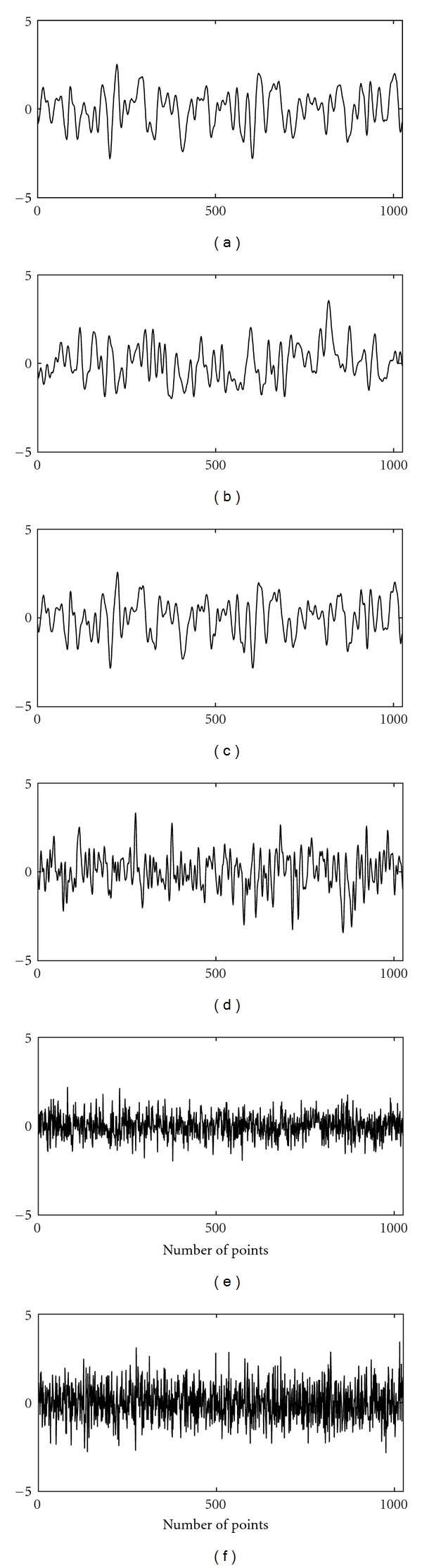
The plots show time series data of (a) 1.5 Hz low-pass filtered WGN, (b) 3 Hz low-pass filtered WGN, (c) 9 Hz low-pass filtered WGN, (d) 30 Hz low-pass filtered WGN, (e) 300 low-pass filtered WGN, and (f) White Gaussian noise.

**Figure 3:**
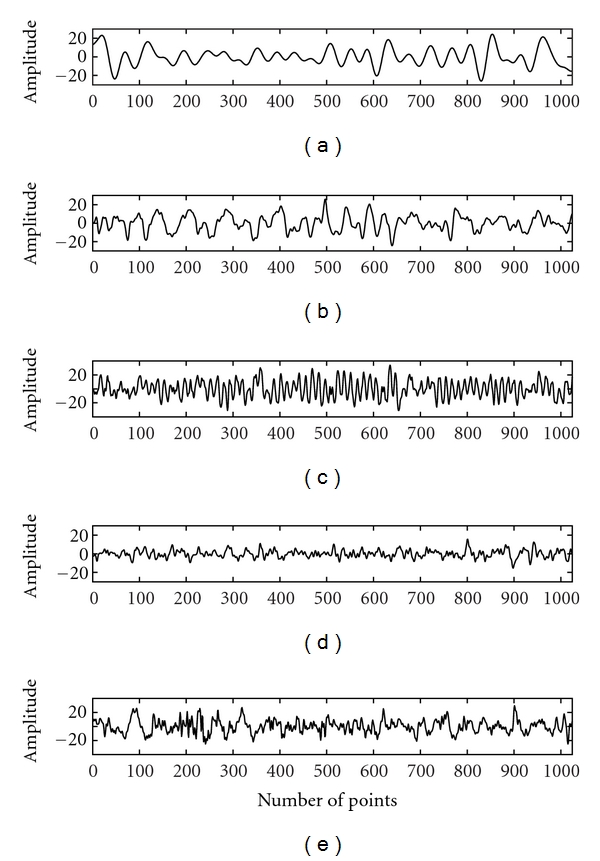
The plots show experimental time series data of various EEG activities: (a) delta EEG activity, (b) theta EEG activity, (c) alpha EEG activity, (d) beta EEG activity, and (e) indeterminate EEG activity.

**Figure 4:**
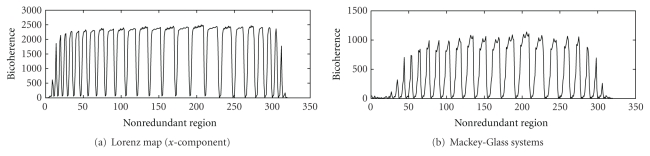
The bicoherence plots of classical chaotic systems: (a) Lorenz map (*x*-component), (b) Mackey-Glass systems.

**Figure 5:**
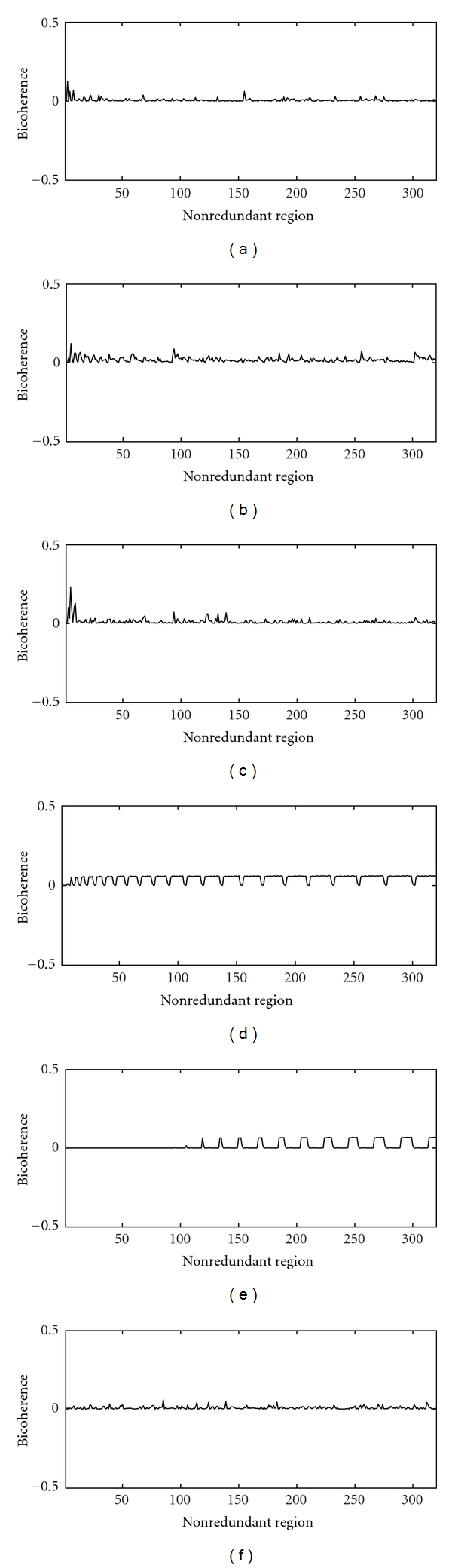
Bicoherence plots of (a) 1.5 low-pass filtered WGN, (b) 3 Hz low-pass filtered WGN, (c) 9 Hz low-pass filtered WGN, (d) 30 Hz low-pass filtered WGN, (e) 300 low-pass filtered WGN, and (f) White Gaussian noise.

**Figure 6:**
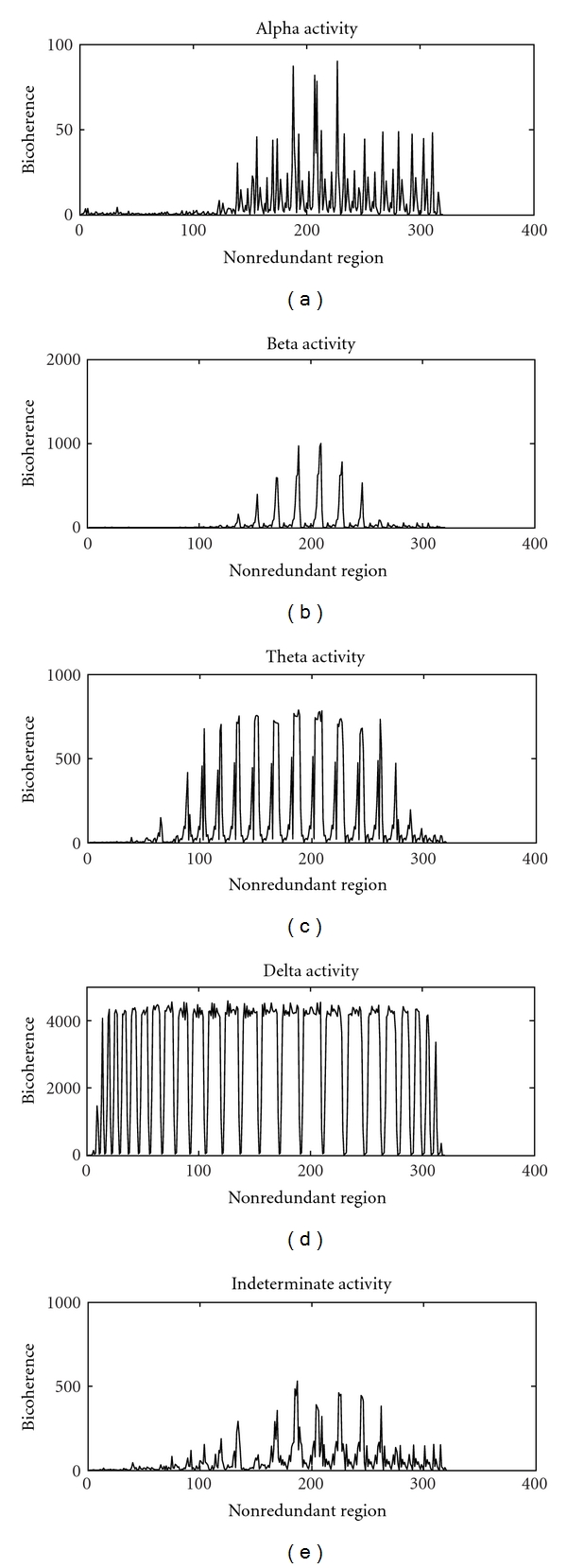
The bicoherence plots of (a) alpha EEG activity, (b) beta EEG activity, (c) theta EEG activity, (d) delta EEG activity, and (e) indeterminate EEG activity.

The Lorenz and the Mackey-Glass systems are examples of classical nonlinear and chaotic systems. These are included in the study for the purpose of comparison with linear systems (WGN, filtered noises) and EEG with a view to demonstrate non-Gaussian and nonlinear characteristics of EEG. The time series of the Lorenz (*x*-component) and the Mackey-Glass systems are shown in Figure 1. The WGN and filtered noises of 1.5 Hz, 3 Hz, 9 Hz, 30 Hz, and 300 Hz used in the study are given in Figure 2. The EEG signals of alpha, beta, theta, delta, and indeterminate activities are shown in Figure 3. We have used 4096 data points of the above time series for estimation of bicoherence and Hinich statistics. The respective bicoherence plots are given in Figures 4–6. The numerical simulation of chaotic time series, WGN, filtered noises, and the method of EEG data acquisition are described in [25].

Table 1 provides probability measure of significance (*χ* ^2^  and *P* values), where the assumption Gaussianity holds good for *P* ≥ 0.5. The results show *P* = 0 for chaotic time series, low-frequency filtered noises (1.5 Hz–30 Hz), and EEG data. Therefore, non-Gaussianity holds good for these time series. The low-frequency filtered noises (1.5 Hz to 30 Hz) are within the bandwidth of normal background EEG activities; therefore, the filtered noises may be considered as spurious processes mimicking EEG rhythms. The results given in Table 1 indicate large differences in the estimated and theoretical interquartile ranges for chaotic time series and the EEG data. In contrast, the estimated and theoretical interquartile ranges (*R*) are relatively small for the WGN and filter noises. As there are no gross differences between them, the assumptions of linearity are not rejected in these data. The large differences in *R*-estimated and *R*-theoretical indicate nonlinearity. Therefore, the normal background EEG rhythms may be treated as nonlinear like chaotic processes. The results clearly establishe non-Gaussian and nonlinear nature of background EEG activities.

## 4. Discussion

Higher-order statistics (spectra) and their application to various signal processing problems are relatively recent. There may be much more information in a stochastic non-Gaussian or deterministic signal than conveyed by its autocorrelation or power-spectral estimates. The higher-order spectra which is defined in terms of the higher-order moments or cumulants of a signal may contain this additional information. The higher-order moments are the natural generalizations of autocorrelation, and the cumulants are specific nonlinear combinations of these moments. The *n*th-order spectrum is defined as the Fourier transform of the *n*th-order cumulant sequence. The test for Gaussianity and linearity is based on the assumption that if the third-order cumulant of a process is zero, then its bispectrum is zero, and hence the bicoherence is also zero. A nonzero bispectrum, therefore, holds good for a non-Gaussian process [23, 30].

For a linear process, the bicoherence is a nonzero constant. If the bispectrum is Gaussian distributed, we know that the squared bispectrum is chi-square distributed with two degrees of freedom. If the estimated bispectrum is zero, then the statistic of bicoherence is a central chi-square random variable with two degrees of freedom. The squared bicoherence is summed over m points in the nonredundant region. Then resulting statistic is chi-square distributed with 2 m degrees of freedom. The statistical test determines the consistency of bicoherence values with a central chi-square distribution. This method is suitable for extracting information about the structure of the signals as it preserves nonminimum phase information. The higher-order spectra carry phase information which is ordinarily suppressed in power spectral estimate. Extensive studies in the field of signal processing have generated information on the input-output relationships of linear systems through autocorrelation and power spectrum. However, no definite information is available on the input-output relationship of nonlinear systems to stochastic excitation and each type of nonlinearity is treated as a special case [23, 30–32].

The present investigation uses the above concepts of higher-order spectral (bicoherence) estimates to obtain Hinich statistics for Gaussianity and linearity. The aim of the study is to contrast the Hinich statistics and bicoherence for well-understood classical chaotic system, WGN, filtered noises, and the normal background EEG activities. The results are shown in Table 1 which provides the *χ* ^2^ values, *P* values, *R*-estimated, *R*-theoretical, and *λ* values. These estimated values of EEG activities may be viewed in reference to obvious nonlinear chaotic series of Lorenz and Mackey-Glass systems and that of filtered noises which fall within its bandwidth.

The bispectral analyses of chaotic systems are found to be characteristically non-Gaussian and nonlinear. The chaos hypothesis in biological systems is based on a finite correlation dimension and a positive dominant Lyapunov exponent. A number of technical problems, however, confound the estimation of these measures. Even the instrumentation at the recording stage may render the application of nonlinear dynamics to these signals invalid. The use of analog filters during signal capture and digital filters in its analysis may account for spurious D_2_ or positive dominant Lyapunov exponent values [25, 33], whereas bispectral methods are robust and least affected by extraneous noises and filters. Here (Table 1), the chaotic time series (Lorenz *x*-component and Mackey-Glass systems) are not only non-Gaussian but also have skewed noncentral distribution (*λ*). In addition, there is a large difference in their *R*-estimated and *R*-theoretical interquartile ranges. The Gaussianity assumption is rejected as the probability of the time series being Gaussian is small (*P* = 0). The linearity hypothesis also cannot be accepted since the differences in the estimated interquartile ranges are much larger than the theoretical values. The squared bicoherence in nonredundant regions are positive and have several peaks (Figure 4); hence, nonlinear coupling is indicated in this systems.

The Hinich statistics (Table 1) also includes the results for WGN, filter noises. The WGN obviously shows highest probability (*P* = 1) as to being Gaussian. Therefore, the results of linearity test are ignored. The stiffly filtered noises (1.5 Hz, 3 Hz, 9 Hz, and 30 Hz) in the low-frequency ranges do depart from Gaussianity (*P* = 0); however, the estimated and theoretical interquartile ranges are very close to each other. The 300 Hz signal is more closer to WGN than the low-frequency signals. Therefore, the Gaussianity test holds good at this high frequency. The linearity hypothesis is also valid for WGN and filtered noises since their estimated and theoretical interquartile ranges (*R*) are close to one another.  Figure 5 shows the estimated mean values of bicoherence over the points in the nonredundant region. The plots do not indicate nonlinear interactions or coupling.

The EEG time series have the lowest probability (*P* = 0) of being non-Gaussian with large differences in their estimated and theoretical interquartile ranges. The high noncentral characteristics (*λ*) are seen only with chaotic and EEG time series. The mean bicoherence of EEG signals are positive and large in the nonredundant regions with several peaks (Figure 6). The bicoherent values are found to be high for delta activity compared to other EEG rhythms. It reflects neocortical forcing for nonlinear coupling by the low-frequency neurons in the deep midbrain and the brain stem structures. We have shown higher bicoherence for all of the normal background EEG rhythms that are associated with a various behavioral states ranging from waking state and alert behavior (beta and indeterminate) to light sleep (alpha and theta) and deep sleep (delta). The normal background EEG activities have bicoherence values ranging from 90 for alpha to 4594.42 for delta. The high-squared bicoherence in nonredundant regions reflect quadratic phase coupling of neuronal ensembles in these conditions. The synchrony of neural discharge (signal morphology) and the non-Gaussian component (frequency coupling) is probably heterogeneous. The squared bicoherence values of normal EEG rhythms (Figures 6(a), 6(b), 6(c), 6(d), and 6(e)) show wide ranging peaks in the nonredundant region. The coupling patterns, therefore, may be different for different EEG activity.

## 5. Conclusion

In our detection of nonlinearity or absence of linear stochastic mechanism, we have shown that the higher-order spectra can reliably distinguish chaotic signals and EEG rhythms from the filtered noises. The filtered noises in the lower passbands show linear stochastic properties in the distribution of their bicoherence values. The results of Hinich statistics and bicoherence estimates indicate that EEG rhythms have similar properties as those of the chaotic time series. The EEG signals are unequivocally non-Gaussian and nonlinear in character. In addition, the bicoherence patterns in the nonredundant regions of the EEG time series are similar to chaotic time series, reflecting quadratic phase coupling. The bispectrum preserves nonminimum phase information of a signal and outputs zero spectrum for linear mechanism. Therefore, the bicoherence statistics of nonredundant region of the spectra (Hinich statistics) may be suitable for detecting hidden structure in signals [25].

## Figures and Tables

**Table 1:** 

Time series	*χ* ^2^	*P*	*R*-estimated	*R*-theoretical	*λ*
Lorenz system	8469.61	0.0	589.87	46.87	301.35
Mackey-Glass system	5765.88	0.00	408.36	38.49	203.02
White Gaussian noise	2.07	1.00	0.08	2.25	0.07
Filtered noise 1.5 Hz	270.08	0.00	16.31	8.67	9.93
Filtered noise 3 Hz	627.06	0.00	46.60	12.99	22.74
Filtered noise 9 Hz	359.46	0.00	19.83	9.95	13.20
Filtered noise 30.0 Hz	120.05	0.00	5.80	5.89	4.40
Filtered noise 300 Hz	55.68	0.21	0.61	4.03	1.84
Alpha EEG activity	9731.75	0.00	390.91	35.75	175.05
Beta EEG activity	34794.08	0.00	1407.53	67.65	628.16
Theta EEG activity	4442.43	0.00	118.94	23.49	75.33
Delta EEG activity	14962.96	0.00	602.83	44.29	269.02
Indeterminate EEG activity	3178.88	0.00	114.14	20.06	54.83

*χ* ^2^ value, *P*: probability of the time series being Gaussian, *R*: interquartile range, *λ*: noncentrality parameter, *d*.*o*.*f* = 271.
